# Vademecum-based approach to multi-scale topological material design

**DOI:** 10.1186/s40323-016-0078-4

**Published:** 2016-08-02

**Authors:** A. Ferrer, J. Oliver, J. C. Cante, O. Lloberas-Valls

**Affiliations:** 1 CIMNE - Centre Internacional de Metodes Numerics en Enginyeria, Campus Nord UPC, Edifici C-1, c/Jordi Girona 1-3, 08034 Barcelona, Spain; 2 Escola Superior d’Enginyeries Industrial, Aeroespacial i Audiovisual de Terrassa, Campus de Terrassa, Edificio TR45. C. Colom, 11 08222 Terrassa, Spain; 3 E.T.S. d’ Enginyers de Camins, Canals i Ports, Technical University of Catalonia, Campus Nord UPC, Edifici C-1, c/Jordi Girona 1-3, 08034 Barcelona, Spain

**Keywords:** Topological optimization, Multiscale material design, Computational Vademecum, Topological derivative, Finite element modelling

## Abstract

The work deals on computational design of structural materials by resorting to computational homogenization and topological optimization techniques. The goal is then to minimize the structural (macro-scale) compliance by appropriately designing the material distribution (microstructure) at a lower scale (micro-scale), which, in turn, rules the mechanical properties of the material. The specific features of the proposed approach are: (1) The cost function to be optimized (structural stiffness) is defined at the macro-scale, whereas the design variables defining the micro-structural topology lie on the low scale. Therefore a coupled, two-scale (macro/micro), optimization problem is solved unlike the classical, single-scale, topological optimization problems. (2) To overcome the exorbitant computational cost stemming from the multiplicative character of the aforementioned multiscale approach, a specific strategy, based on the consultation of a discrete material catalog of micro-scale optimized topologies (Computational Vademecum) is used. The Computational Vademecum is computed in an offline process, which is performed only once for every constitutive-material, and it can be subsequently consulted as many times as desired in the online design process. This results into a large diminution of the resulting computational costs, which make affordable the proposed methodology for multiscale computational material design. Some representative examples assess the performance of the considered approach.

## Background

In the last decades, topological structural optimization has gained considerable importance in the Computational Mechanics field. Besides the increasing interest of the scientific community, practical applications have been accomplished in the aeronautical [1], automotive and civil engineering industry. In fact, topology optimization tools can be found in more than thirty commercial software packages [2], e.g. Abaqus [3], Altair HyperWorks [4].

After the groundbreaking contribution in [5], a density-like approach, termed the solid isotropic material with penalization (SIMP) method, has been successfully developed during the last years. Unfortunately, some numerical problems may occur, like checkerboard and mesh-dependency. Furthermore, artificial parameters must be provided.

Alternatively, other approaches, such as evolutionary structural optimization (ESO/BESO), have been proposed [6]. Even though a pseudo-density does not need to be defined, heuristic criteria is used to find possible sub-optimal solutions.

More recently, shape sensitivity has become a successful topological optimization tool. Initially, in [7], the so-called shape optimization theory was introduced by Allaire. Within this theory, the topology optimization problem is solved by means of a level set algorithm and a Hamilton–Jacobi evolution equation. Nevertheless, in order to remove material, a topology with holes must be provided as an initial guess. Such conditioning leads to specific optimal topologies. In addition, from the work in [8], the topological derivative mathematical concept has been recently used for structural topology optimization. Both shape optimization and asymptotic expansion theories are essential for the closed-form expression of the topological derivative. Its success is due to the analytical expressions that measure an specific shape functional sensitivity when an inclusion is introduced into a fixed domain. Later studies have been consolidated in the reference book [9]. Additionally, in [10], a level set algorithm is proposed taking advantage of the topological derivative theory as a gradient of steepest descent algorithm. It is worth noting that this last approach is exempt from artificial parameters.

Bearing this in mind, the same methodology can be used for material design [11] besides structural optimization. Computational multi-scale approaches provide an appropriate framework to achieve it. Either asymptotic multi-scale [12] or variational multi-scale theories [13] have been extensively used in the recent years. Although the former is based on analytical expansion theories, the latter seems to fit more naturally into the FEM context, specially due to its variational framework. In practice, they lead to a $\text {FE}{}^{2}$ problem where standard FEM discretizations of the elastic equations are considered at both the macro and micro-scales.

Apart from [11], other works (cf. [14–16]) have recently studied topological optimization in a multi-scale context, the main drawback being the expensive computational multi-scale problem to be faced. In consequence, and in order to keep bounded the computational cost, either the micro-structure topology is kept constant over the whole domain or very coarse discretizations of this microstructure can only be considered.

In this work, an alternate directions algorithm is proposed as in [12]. Then, inspired in the Computational Vademecum concept [17], the computationally expensive construction of a catalog of optimal material designs is made of-line, and only a trivial selection of the optimal micro-structure is done in the on-line process. As in PGD [17], or POD strategies [18, 19], a considerable reduction on the computational cost is obtained. To validate this approach, some numerical examples are shown.

The paper is organized as follows: first, in “Multi-scale topology optimization framework” section, the variational multi-scale theory is presented and its main features are summarized. Additionally, the topological derivative concept on the micro-scale is introduced and the useful final expression is shown. A level-set based algorithm is described and some examples are presented. In “Multi-scale topology optimization problem” section, the formulation of the target problem is stated and conveniently reformulated. A preliminary study of the problem complexity is also presented. In “Vademecum-based computational cost reduction” section, the Computational Vademecum is computed and some examples are shown. In addition, the homogenized constitutive tensor distribution is represented. In “Numerical algorithm” section, an alternate directions algorithm is conceptually described and some numerical examples are solved in “Numerical examples” section. Conclusions and future perspectives are summarized in “Concluding remarks” section.

## Multi-scale topology optimization framework

### Multi-scale problem

In some cases, phenomenological constitutive laws do not suffice to represent the micro-scale behavior and predict the macroscopic material properties. On one hand, heterogeneities could only be captured in the finite element context with unaffordable fine meshes. On the other hand, highly demanding applications require better accuracy of the constitutive modeling.

**Fig. 1:**
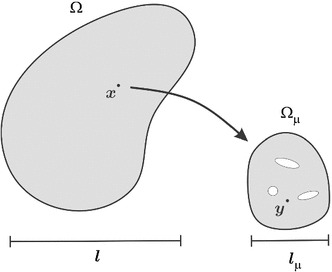
Macroscopic and microscopic domains

To overcome this difficulty, multi-scale techniques, based on homogenization theories, aim at representing heterogeneities of the small length scale $l_{\mu }$ by taking mean values of the micro-scale material properties. Each homogeneous region of the continuum macroscopic domain, represented by the corresponding integration/sampling point, retrieves the constitutive law from the average response of a representative microscopic domain, i.e. the representative volume element (RVE) (cf. Fig.  1).

Different theories have been developed in order to set up the corresponding mathematical framework [12, 13]. Asymptotic expansion and variational multi-scale may be nowadays the most successful approaches in the computational mechanics community. Even though asymptotic expansion is a rigorous mathematical theory and has been used for a long time, variational multi-scale theory seems to be easier to generalize and extend to non-linear problems. Furthermore, variational approaches usually fit more naturally in the context of finite element methods. In our study, this last approach will be used. For more information, the reader is referred to the works in [13].

#### Multi-scale variational framework

Firstly, this framework sets that the strain on the micro-scale, $\epsilon _{\mu }(x)$, is defined by the sum of the macro-scale strain, $\epsilon (x)$, and a fluctuating strain $\tilde{\epsilon }_{\mu }(x,y)$. Secondly, as an axiom, a zero mean value on the micro-scale is established. That is,

1 $$\begin{aligned}&\epsilon _{\mu }(x,y)=\epsilon (x)+\tilde{\epsilon }_{\mu }(x,y),\end{aligned}$$

2 $$\begin{aligned}&\int _{\Omega _{\mu }}\tilde{\epsilon }_{\mu }(x,y)=0. \end{aligned}$$

As a consequence of this, the macro-scale strain becomes the volume average of the micro-scale strain, i.e.

3 $$\begin{aligned} \epsilon (x)={\frac{1}{\Omega _{\mu }}}\int _{\Omega _{\mu }} \epsilon _{\mu }(x,y). \end{aligned}$$

At this time, the functional spaces of the macroscopic and fluctuation strains can be defined as

4 $$\begin{aligned} \mathbb {V}_{\epsilon }= & {} \{\epsilon (x)\in T^{2}(\mathbb {R}^{d},\mathbb {R})\},\end{aligned}$$

5 $$\begin{aligned} \mathbb {V}_{\tilde{\epsilon }_{\mu }}= & {} \left\{ \tilde{\epsilon }_{\mu }(x,y)|\int _{\Omega _{\mu }} \tilde{\epsilon }_{\mu }(x,y)=0\right\} \end{aligned}$$

being $T^{2}(\mathbb {R}^{d},\mathbb {R})$ the symmetric second order tensor space. Thus, the micro-scale strain space function becomes simply

6 $$\begin{aligned} \mathbb {V}_{\epsilon _{\mu }}= & {} \{\epsilon _{\mu }(x,y)|\epsilon _{\mu }(x)= \epsilon (x)+\tilde{\epsilon }_{\mu }(x,y)\}, \end{aligned}$$

with $\epsilon \in \mathbb {V}_{\epsilon }$ and $\tilde{\epsilon }_{\mu }\in \mathbb {V}_{\tilde{\epsilon }_{\mu }}$. The definition of these spaces plays a key role on the Hill-Mandel Principle. For more details, see [13].

#### Hill- Mandel principle

The Hill-Mandel principle postulates that the internal energy of a macroscopic point should be equal to the volume average of the microscopic internal energy [13], that is

7 $$\begin{aligned} \sigma :\delta \epsilon =\frac{1}{\Omega _{\mu }}\int _{\Omega _{\mu }} \sigma _{\mu }:\delta \epsilon _{\mu }\forall \delta \epsilon _{\mu } \in \mathbb {V}_{\epsilon _{\mu }}. \end{aligned}$$

where $\sigma$ is the macro-scale stress tensor and $\sigma _{\mu }$ is the micro-scale stress tensor.

According to (6) a variation of the microscopic strain implies a variation on the macroscopic strain and a variation on the microscopic fluctuation, i.e.,

8 $$\begin{aligned} \delta \epsilon _{\mu }=\delta \epsilon +\delta \tilde{\epsilon }_{\mu }. \end{aligned}$$

Since the Hill-Mandel principle holds for all $\delta \epsilon _{\mu }\,{\in }\,\mathbb {V}_{\epsilon _{\mu }}$, it also holds for:

- First, $\delta \tilde{\epsilon }_{\mu }=0\Rightarrow \delta \epsilon _{\mu }=\delta \epsilon$ and, as a result, (7) takes the following form,
  9 $$\begin{aligned} \sigma :\delta \epsilon =\frac{1}{\Omega _{\mu }}\int _{\Omega _{\mu }} \sigma _{\mu }:\delta \epsilon \forall \delta \epsilon \,{\in }\,\mathbb {V}_{\epsilon }. \end{aligned}$$

Applying the fundamental lemma of calculus of variations, the first consequence of the Hill-Mandel principle is that the macroscopic stress tensor is the average of the micro-scale stress tensor over the whole RVE,

10 $$\begin{aligned} \sigma (x)={\frac{1}{\Omega _{\mu }}}\int _{\Omega _{\mu }} \sigma _{\mu }(x,y). \end{aligned}$$

- Second, $\delta \epsilon =0\Rightarrow \delta \epsilon _{\mu }=\delta \tilde{\epsilon }_{\mu }$, therefore (7) becomes
  11 $$\begin{aligned} \sigma :\delta \epsilon =\frac{1}{\Omega _{\mu }}\int _{\Omega _{\mu }} \sigma _{\mu }:\delta \tilde{\epsilon }_{\mu }=0 \forall \delta \tilde{\epsilon }_{\mu }\,{\in }\, \mathbb {V}_{\tilde{\epsilon }_{\mu }}. \end{aligned}$$

Therefore, the second consequence of the Hill-Mandel principle is the equilibrium equation of the RVE

12 $$\begin{aligned} \int _{\Omega _{\mu }}\sigma _{\mu }:\delta \tilde{\epsilon }_{\mu }=0 \forall \delta \tilde{\epsilon }_{\mu }\in \mathbb {V}_{\tilde{\epsilon }_{\mu }}. \end{aligned}$$

This result points out that the fluctuations do not produce work, i.e. they have zero internal energy.

#### Equilibrium equation and micro-cell boundary conditions

For the case of linear elastic behavior in the micro-scale, the constitutive equation takes the form

13 $$\begin{aligned} \sigma _{\mu }(x,y)=\mathbb {\mathbb {C}}_{\mu }(x,y):\epsilon _{\mu }(x,y). \end{aligned}$$

Therefore Eq. (12) is rewritten as

14 $$\begin{aligned} \int _{\Omega _{\mu }}\epsilon _{\mu }:\mathbb {\mathbb {C}}_{\mu }: \delta \tilde{\epsilon }_{\mu }=0\forall \delta \tilde{\epsilon }_{\mu } \in \mathbb {V}_{\tilde{\epsilon }_{\mu }}, \end{aligned}$$

and considering (1) the equilibrium equation results

15 $$\begin{aligned} \int _{\Omega _{\mu }}\tilde{\epsilon }_{\mu }:\mathbb {\mathbb {C}}_{\mu }: \delta \tilde{\epsilon }_{\mu }=-\int _{\Omega _{\mu }}\epsilon : \mathbb {\mathbb {C}}_{\mu }:\delta \tilde{\epsilon }_{\mu }\forall \delta \tilde{\epsilon }_{\mu }\in \mathbb {V}_{\tilde{\epsilon }_{\mu }}. \end{aligned}$$

After applying the Gauss theorem, (2) can be written as,

16 $$\begin{aligned} \int _{\Omega _{\mu }}\tilde{\epsilon }_{\mu }=\int _{\Omega _{\mu }} \nabla ^{s}\tilde{u}_{\mu }=\int _{\partial \Omega _{\mu }}\tilde{u}_{\mu } \otimes _{s}n=0, \end{aligned}$$

where $\tilde{u}_{\mu }$ is the fluctuation displacement field. In fact, integrating (1) the micro-scale displacement $u_{\mu }$ fulfills

17 $$\begin{aligned} u_{\mu }(x,y)=\epsilon (x)y+\tilde{u}_{\mu }(x,y). \end{aligned}$$

So that, defining the space function of the fluctuation displacement as

18 $$\begin{aligned} \mathbb {V}_{\tilde{u}_{\mu }}=\left\{ \tilde{u}_{\mu }(x,y)| \int _{\partial \Omega _{\mu }}\tilde{u}_{\mu }\otimes _{s}n=0 \right\} , \end{aligned}$$

the equilibrium Eq. (15) becomes

19 $$\begin{aligned} \int _{\Omega _{\mu }}\nabla ^{s}\tilde{u}_{\mu }: \mathbb {\mathbb {C}}_{\mu }:\nabla ^{s}\delta \tilde{u}_{\mu }= -\int _{\Omega _{\mu }}\epsilon :\mathbb {\mathbb {C}}_{\mu }: \nabla ^{s}\delta \tilde{u}_{\mu }\forall \delta \tilde{u}_{\mu } \in \mathbb {V}_{\tilde{u}_{\mu }}. \end{aligned}$$

Therefore, a micro-scale problem involves solving a classical weak formulation of an equilibrium problem subject to the constraint (18). There are different approaches to satisfy this boundary condition of the RVE. In literature, see [13], the most frequently used are Taylor, linear, periodic and minimum condition.

**Fig. 2:**
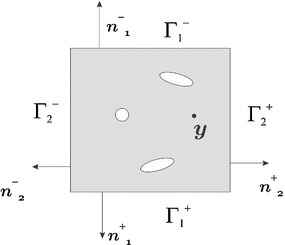
RVE periodic domain, square cell

Materials with periodic micro-structures are suitable for periodic conditions. For some specific micro-scale geometries like square cells (hexagonal cells, and others is similar), the boundary can be divided in two parts $\Gamma _{1}$ and $\Gamma _{2}$ (see Fig. 2) with outward unit normal such that,

20 $$\begin{aligned} n_{1}^{+}=-\mathbf { n }_{1}^{-}; \quad \mathbf {\mathrm { n } _{\mathrm {2}}^{\mathrm {+}}}=-\mathbf { n }_{2}^{-}. \end{aligned}$$

Thus, condition (18) may be written as

21 $$\begin{aligned} \int _{\partial \Omega _{\mu }}\tilde{u}_{\mu }\otimes _{s}n= & {} \int _{\Gamma _{1}^{+}}\tilde{u}_{\mu }^{(1)^{+}}\otimes _{s}n_{1}^{+}+ \int _{\Gamma _{2}^{+}}\tilde{u}_{\mu }^{(2)^{+}}\otimes _{s}n_{2}^{+}\nonumber \\&+\int _{\Gamma _{1}^{-}}\tilde{u}_{\mu }^{(1)^{-}}\otimes _{s}n_{1}^{-} + \int _{\Gamma _{2}^{-}}\tilde{u}_{\mu }^{(2)^{-}}\otimes _{s}n_{2}^{-} =0, \end{aligned}$$

where $\tilde{u}_{\mu }^{(1)^{+}}$ is the fluctuation on $\Gamma _{1}^{+}$ and $\tilde{u}_{\mu }^{(2)^{+}}$ is the fluctuation on $\Gamma _{2}^{+}$. Equivalently, for $\Gamma _{1}^{-}$ and $\Gamma _{2}^{-}$.

Taking into account (20),

22 $$\begin{aligned} \int _{\partial \Omega _{\mu }}\tilde{u}_{\mu }\otimes _{s}n = \int _{\Gamma _{1}^{+}}(\tilde{u}_{\mu }^{(1)^{+}}-\tilde{u}_{\mu }^{(2)^{+}}) \otimes _{s}n^{+}+ \int _{\Gamma _{1}^{-}}(\tilde{u}_{\mu }^{(1)^{-}}-\tilde{u}_{\mu }^{(2)^{-}}) \otimes _{s}n^{-}=0. \end{aligned}$$

Equation (21) is satisfied using periodic boundary conditions imposing that

23 $$\begin{aligned} \tilde{u}_{\mu }^{(1)^{+}}= & {} \tilde{u}_{\mu }^{(2)^{+}}\end{aligned}$$

24 $$\begin{aligned} \tilde{u}_{\mu }^{(1)^{-}}= & {} \tilde{u}_{\mu }^{(2)^{-}} \end{aligned}$$

#### The homogenized constitutive tensor

Homogenization of the constitutive tensor is an essential part of the computational multi-scale approach. As in other fields of mechanics, its definition is given by

25 $$\begin{aligned} \mathbb {C}^{h}:=\frac{\partial \sigma }{\partial \epsilon }= \frac{1}{\Omega _{\mu }}\int _{\Omega _{\mu }}\frac{\partial \sigma _{\mu }}{\partial \epsilon } =\frac{1}{\Omega _{\mu }}\int _{\Omega _{\mu }}\mathbb {C_{\mu }}: \frac{\partial \epsilon _{\mu }}{\partial \epsilon }, \end{aligned}$$

where Eq. (10) and linear elastic hypothesis have been used.

Taking derivatives of (1) w.r.t. the macroscopic strain,

26 $$\begin{aligned} \frac{\partial \epsilon _{\mu }}{\partial \epsilon }= \mathbb {I}+\frac{\partial \nabla ^{s}\tilde{u}_{\mu }}{\partial \epsilon }= \mathbb {I}+\mathbb {A}(y) \end{aligned}$$

being $\mathbb {I}$ the symmetric fourth-order identity tensor and $\mathbb {A}(y)$ the fourth order localization tensor. From (19), there is a linear dependency between $\nabla ^{s}\tilde{u}_{\mu }$ and $\epsilon$, thus,

27 $$\begin{aligned} \nabla ^{s}\tilde{u}_{\mu }=\mathbb {A}(y):\epsilon . \end{aligned}$$

Due to linearity, a straightforward calculus of the localization tensor is performed by computing $\nabla ^{s}\tilde{u}_{\mu }$ from (19) for each canonical base.

Consequently, the homogenized constitutive tensor takes the form

28 $$\begin{aligned} \mathbb {C}^{h}&={\displaystyle \dfrac{1}{\Omega _{\mu }}\int _{\Omega _{\mu }}\mathbb {C_{\mu }}+ \dfrac{1}{\Omega _{\mu }}\int _{\Omega _{\mu }}\mathbb {C_{\mu }}: \mathbb {A}(y)}\nonumber \\&=\bar{\mathbb {C}}+\tilde{\mathbb {C}} \end{aligned}$$

$\mathbb {\bar{C}}$ and $\tilde{\mathbb {C}}$ denoting, respectively, the volume average elastic micro-scale constitutive tensor and the fluctuation contribution of the homogenized constitutive tensor.

Finally, due to linearity, the macroscopic constitutive equation can be written as,

29 $$\begin{aligned} \sigma =\mathbb {C}^{h}:\epsilon . \end{aligned}$$

### Topological derivative at the micro-scale

Recently, topological derivative has emerged as a useful tool for structural topology optimization [10, 12, 20, 21] and material design [11, 22]. Sokolowski and Zochowski, in [8], set up the foundation of the topological derivative theory, and some years later, such theory consolidates in the reference book [9].

In the topological derivative theory context, a closed formula is obtained as the sensitivity of a shape functional when an inclusion on the domain is introduced. Its derivation is based primarily on the concepts of shape optimization and topological asymptotic expansion as follows.

#### Topological derivative of the homogenized constitutive tensor

First, an unperturbed RVE domain, $\Omega _\mu$ is introduced . From the strong form of the problem (19), the microscopic equilibrium equations can be written as,

30 $$\begin{aligned} \left\{ \begin{array}{rclcl} \nabla \cdot \sigma _{\mu }(\widetilde{u}_{\mu }) &{} = &{} 0 &{} \quad {\text {in}} &{} \Omega _{\mu },\\ \sigma _{\mu }(\widetilde{u}_{\mu }) &{} = &{} \mathbb {C_{\mu }}:\nabla ^{s}\widetilde{u}_{\mu },\\ \tilde{u}_{\mu }^{(1)} &{} = &{} \tilde{u}_{\mu }^{(2)} &{} \quad \text {on} &{} \partial \Omega _{\mu }. \end{array}\right. \end{aligned}$$

**Fig. 3:**
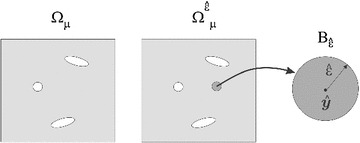
Micro-scale elastic problem on the perturbed domain

Subsequently, the elastic problem on the perturbed domain is presented. First, a circular hole $B_{\hat{\epsilon }}$ of radius ${ \hat{\epsilon }}$ and center $\hat{y}$ is set in the domain ${\Omega _\mu }$, and then an inclusion of the same shape but with different material properties is introduced, see Fig. 3 . For the case of an actual void created in the domain $\Omega$, the reader is referred to the work in [9].

To account for this case the superscript notation $\left( \bullet \right) ^{ \hat{\epsilon }}$ is introduced. Consequently, the strong form of the equilibrium equation on the perturbed domain $\Omega _{\mu }^{{ \hat{\epsilon }}}$ reads as follows:

31 $$\begin{aligned} \left\{ \begin{array}{rclcl} \nabla \cdot \sigma _{{{\mu } }}^{{{ \hat{\epsilon }}}}(\tilde{u}_{{{\mu }}}^{{ \hat{\epsilon }}}) &{} = &{} 0 &{} \quad {\text {in}} &{} \Omega _{\mu }^{{ \hat{\epsilon }}},\\ \sigma _{{{\mu } }}^{{ \hat{\epsilon }}}(\tilde{u}_{{{\mu }}}^{{ \hat{\epsilon }}}) &{} = &{} \gamma _{{ \hat{\epsilon }}}\mathbb {C_{\mu }}:\nabla ^{s}\tilde{u}_{{{\mu }}}^{{ \hat{\epsilon }}},\\ \left( \tilde{u}_{{{\mu }}}^{{ \hat{\epsilon }}}\right) ^{(1)} &{} = &{} \left( \tilde{u}_{{{\mu }}}^{{ \hat{\epsilon }}}\right) ^{(2)} &{} \quad \text {on} &{} \partial \Omega _{\mu }^{{ \hat{\epsilon }}}.\\ \llbracket \tilde{u}_{{{\mu }}}^{{ \hat{\epsilon }}}\rrbracket &{} = &{} 0 &{} \quad \text {on} &{} \partial B_{{ \hat{\epsilon }}},\\ \llbracket \sigma _{{{\mu } }}^{{ \hat{\epsilon }}}(\tilde{u}_{{{\mu }}}^{{ \hat{\epsilon }}})\rrbracket \cdot n &{} = &{} 0 &{} \quad \text {on} &{} \partial B_{{ \hat{\epsilon }}}, \end{array}\right. \end{aligned}$$

where $\gamma _{ \hat{\epsilon }}$ (the *contrast parameter*) sets the material stiffness value at the inclusion, $B_{ \hat{\epsilon }}$, and at the unperturbed material, $\Omega _{\mu }\backslash \bar{B}_{ \hat{\epsilon }}$, as:

32 $$\begin{aligned} {\scriptstyle }\gamma _{ \hat{\epsilon }}=\left\{ \begin{array}{ll} 1 &{} \quad \text {in} ~~ \Omega _{\mu }\backslash \bar{B}_{ \hat{\epsilon }},\\ \gamma &{} \quad \text {in}~~ B_{ \hat{\epsilon }}. \end{array}\right. \end{aligned}$$

Bearing this in mind, an asymptotic expansion of the homogenized constitutive tensor $\mathbb {C}^{h}$ is proposed as,

33 $$\begin{aligned} \mathbb {C}_{ \hat{\epsilon }}^{h}=\mathbb {C}^{h}+f({ \hat{\epsilon }})D_{T}\mathbb {C}^{h}+o(f({ \hat{\epsilon }})) \end{aligned}$$

where $\mathbb {C}^{h}$ and $\mathbb {C}_{ \hat{\epsilon }}^{h}$ stand for the homogenized constitutive tensor (25) of problems (30) and (31) respectively. For the considered case, $f({ \hat{\epsilon }})$ takes the following value:

34 $$\begin{aligned} f({ \hat{\epsilon }})= & {} \frac{\pi { \hat{\epsilon }}^{2}}{|\Omega _{\mu }|}. \end{aligned}$$

Then, after some mathematical derivations, the topological derivative of the homogenized constitutive tensor, $D_{T}\mathbb {C}^{h}$, yields a fourth-order tensor, whose final expression, see [9], for plane stress cases reads (in Cartesian coordinates and using indicial notation):

35 $$\begin{aligned} \left( D_{T}\mathbb {C}^{h}\right) _{ijkl}=\sigma _{{{\mu } }}(\widetilde{u}_{\mu _{ij}}):\mathbb {H}:\sigma _{{{\mu } }}(\widetilde{u}_{\mu _{kl}}), \end{aligned}$$

where $\mathbb {H}$ is also a fourth-order tensor, depending on the material properties, whose expression is given by:

36 $$\begin{aligned} \mathbb {H}=-\frac{1}{E_{\mu }}\left( \frac{1-\gamma }{1+\alpha \gamma }\right) \left[ 4\mathbb {I}- \frac{1-\gamma (\alpha -2\beta )}{1+\beta \gamma }(I\otimes I)\right] \end{aligned}$$

being

$$\begin{aligned} \alpha =\frac{1+\nu _{\mu }}{1-\nu _{\mu }}, \quad \beta =\frac{3-\nu _{\mu }}{1+\nu _{\mu }}. \end{aligned}$$

In Eq. (36) $E_{\mu }$ and $\nu _{\mu }$ are the Young modulus and the Poisson ratio of the micro-scale material. Notice that the topological derivative (35) is a tensor field defined point-wise, which depends only on the solution of the unperturbed problem, the micro-scale material parameters and the contrast parameter of the inclusion. For a complete and rigorous derivation of the expression in (35) the reader is referred to [11].

#### Level set algorithm

An important implication of the topological derivative concept is that the homogenized constitutive tensor sensibility to the introduction of an inclusion may be determined. This allows using this sensibility as the driving-force in a topological optimization problems (cf. [10]).

Briefly, the minimization problem is stated as

37 $$\begin{aligned} \underset{\chi }{\text {minimize}}&J(\chi ) \end{aligned}$$

where $\chi$ is the characteristic function, stating the distribution of material and voids (or small-stiffness inclusions), and $J(\chi )$ is the cost function.

Defining a $C^{1}(\Omega )$ nodal level-set field $\psi$, over the domain, such that,

38 $$\begin{aligned} {\chi }={\left\{ \begin{array}{ll} \begin{array}{c@{\quad }c} 1 \quad &{} \psi <0,\\ 0 \quad &{} \psi >0. \end{array}\end{array}\right. } \end{aligned}$$

problem (37) yields

39 $$\begin{aligned} \underset{\psi }{\text {minimize}}&J(\chi (\psi )) \end{aligned}$$

and, consequently, the level-set field $\psi$ becomes the new unknown variable. To solve Eq. (39) a slerp-type algorithm, see [10], is proposed through the recursive formula:

40 $$\begin{aligned} \psi _{n+1}=\frac{1}{\sin \theta _{n}}[\sin ((1-\kappa _{n}) \theta _{n})\psi _{n}+\sin (\kappa _{n}\theta _{n})\frac{g_{n}}{||g_{n}||_{L^{2}}}], \end{aligned}$$

being $\kappa _{n}\in [0,1]$ a line search-like parameter, $g_{n}$ the topological derivative and $\theta _{n}$ the angle between $\psi _{n}$ and $g_{n}$ which is written as

41 $$\begin{aligned} \theta _{n}= & {} \text {acos}\left[ \frac{(\psi _{n},g_{n})}{||\psi ||_{L^{2}} ||g_{n}||_{L^{2}}}\right] . \end{aligned}$$

providing the converge criterion when it is smaller than a certain tolerance. The iterative procedure in equations (40) and (41) can be regarded as an update of the level set, $\psi _{n+1}$, to match the topological derivative $g_{n}$ in the unit sphere (slerp), along a line-search step of parameter $\kappa _{n}$, which, in turn, is chosen to provide the maximum decrease of the objective function $J(\chi (\psi ))$.

### Representative examples

To illustrate the methodology proposed above some examples are presented next. The RVE structural compliance is aimed at being minimized through the following minimization problem:

42 $$\begin{aligned} \begin{array}{cc} \underset{\chi _{\mu }}{\text {minimize}} \mathbb {{ \sigma }}: \mathbb {C}_{h}^{-1}(\chi _{\mu }):{ \sigma }\\ \text {subjected to} :\displaystyle {\int _{\Omega _{\mu }}\chi _{\mu }=V_{\mu }} \end{array} \end{aligned}$$

where $V_{\mu }$ is the RVE solid-volume, ${ \sigma }$ stands for the unit norm macroscopic stress tensor, here considered the driving force for the homogenization problem, and $\mathbb {C}_{h}$ is the homogenized constitutive tensor.

Following the work in [20, 23, 24] an augmented Lagrangian algorithm is used to solve the unconstrained problem (42), which can be rewritten as

43 $$\begin{aligned} \underset{\lambda }{\text {max.}}&\underset{\chi _{\mu }}{\text {min.}}&{ \sigma }:\mathbb {C}_{h}^{-1}(\chi _{\mu }):{ \sigma }+\lambda \left( \int _{\Omega _{\mu }}\frac{\chi _{\mu }}{V_{\mu }}-1\right) +\frac{1}{2}\rho \left( \int _{\Omega _{\mu }}\frac{\chi _{\mu }}{V_{\mu }}-1\right) ^{2}. \end{aligned}$$

where $\rho$ is the penalty parameter. Straightforwardly, the topological derivative is computed as

44 $$\begin{aligned} g(\chi _{\mu })= & {} -\mathbb {C}_{h}^{-1}(D_{T}\mathbb {C}_{h})\mathbb {C}_{h}^{-1}+ \lambda \frac{1}{V_{\mu }}+\rho \left( \int _{\Omega _{\mu }}\frac{\chi _{\mu }}{V_{\mu }}-1 \right) \frac{1}{V_{\mu }}. \end{aligned}$$

Finally, problem (43) is solved using algorithm (40) and the augmented Lagrangian-type update of lambda:

45 $$\begin{aligned} \lambda _{n+1}= & {} \lambda _{n}+\rho \left( \int _{\Omega _{\mu }}\frac{\chi _{\mu }}{V_{\mu }}-1\right) . \end{aligned}$$

As a matter of example, three different cases are computed now. In all of them, the initial topology is selected as in [11], the initial Lagrange multiplier is $\lambda _{0}=0$, the final solid-volume is $V_{\mu }=0.6$, the elastic parameters are $E_{\mu }=1,$ $\nu _{\mu }=0.3$, the contrast parameter is $\gamma =0.001$ and the penalty is chosen $\rho =1$. The algorithm stops when $\theta <1^{0}$ and the volume constraint tolerance $Tol<0.001$.

**Fig. 4:**
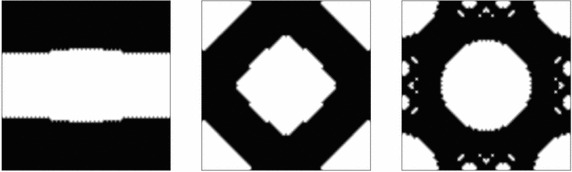
Horizontal, shear and bulk stress-state optimal RVE topologies

**Fig. 5:**
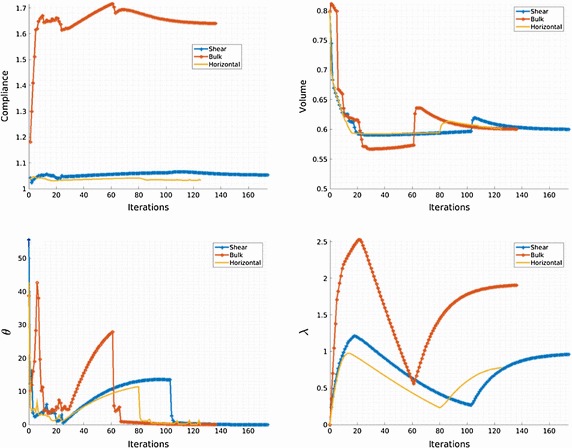
Relevant information evolution along the iterative process

The selection of the macroscopic unit stress ${ \sigma }$ defines the case of study. That is, in Voight notation:

- Uniaxial horizontal stress-state: $${\sigma }=\left[ \begin{array}{c} 1\\ 0\\ 0 \end{array}\right] $$
- Shear stress-state: $${ \sigma }=\left[ \begin{array}{c} 0\\ 0\\ 1 \end{array}\right] $$
- Bulk stress-state: $${ \sigma }=\frac{1}{\sqrt{2}}\left[ \begin{array}{c} 1\\ 1\\ 0 \end{array}\right] $$

The obtained optimal topologies are presented in Fig. 4. All the relevant information is summarized in Fig. 5. Notice that the objective function oscillates during the iterations, mimicking the saddle point feature of the problem (43). It should be also remarked that the algorithm is capable of conveniently adding and subtracting material along the iterations. In all cases less than 10 min of computation are needed with a standard PC (3.40GHz processor in a 64-bit architecture) in a vectorized Matlab$^{\copyright }$ environment.

**Fig. 6:**
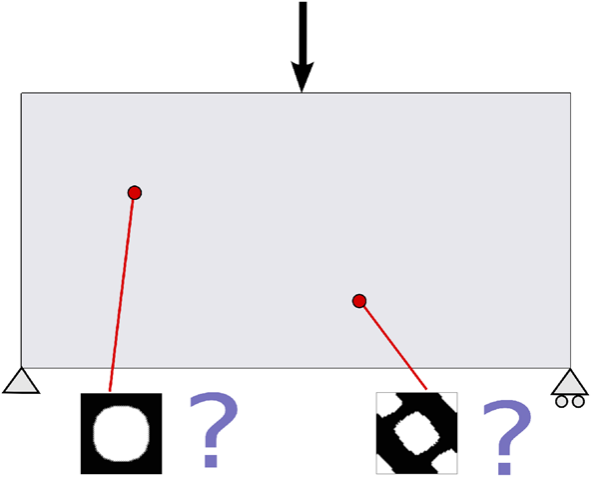
Multi-scale topology optimization problem

## Multi-scale topology optimization problem

Once the computational multi-scale homogenization framework has been introduced and the micro-structure topology optimization method has been described, next step is to use these ingredients for material design purposes. In other words, to determine the optimal micro-structure topology at every point of the macro-structure domain in order to achieve a functional goal at the macro-scale level as it is sketched in Fig. 6.

### Formulation

The classical topological optimization problem in structural analysis refers to the minimum compliance (or maximum stiffness) design. Here the classical single-scale problem of determining the optimum distribution of a certain material mass at the macro-scale (or structural scale), to achieve the minimum compliance of the resulting structure [5], is reformulated as a two-scale problem in the following sense: the goal is the optimal distribution of a given material mass, *but now at the micro-scale* level for every structural point (given the shape and topology at the structural scale).

The problem is mathematically stated through:

46 $$\begin{aligned} \begin{array}{cc} \underset{\chi _{\mu }}{\text {minimize}} &{} \displaystyle {\int _{\Omega }\sigma (\chi _{\mu }):\mathbb {C}_{h}^{-1}(\chi _{\mu }):\sigma (\chi _{\mu })}\\ \text {subjected to:} &{} \displaystyle {\frac{\int _{\Omega _{\mu }}\chi _{\mu }}{V_{\mu }}-1\le 0}, \end{array} \end{aligned}$$

where the macroscopic stresses, $\sigma ,$ fulfill Eq. (29) and are the solution of an standard linear elasticity problem. From the optimization point of view, problem (46) may be appropiatly rewritten as,

47 $$\begin{aligned} \begin{array}{cc} \underset{\sigma ,\chi _{\mu }}{\text {minimize}} &{} \displaystyle {\int _{\Omega }\sigma :\mathbb {C}_{h}^{-1}(\chi _{\mu }):\sigma }\\ \text {subjected to:} &{} \displaystyle {\frac{\int _{\Omega _{\mu }}\chi _{\mu }}{V_{\mu }}}-1\le 0,\\ &{} \nabla \cdot \sigma =\rho b,\\ &{} \text {+ Boundary conditions.} \end{array} \end{aligned}$$

In Eq. (47), $\chi _{\mu }\in \left\{ 0,1\right\}$, refers to the characteristic function at the RVE, whose optimal spatial distribution (defining the topology of the RVE) is aimed at being obtained, and $V_{\mu }$ refers to the “measure” (area or volume) of the RVE. In this respect the following aspects, specific for this multi-scale problem, have to be highlighted:

1. The objective function to be minimized is highly nonlinear and defined at the macro-scale level.
2. The design variables (the values of the characteristic function ${{\chi }}_{\mu }$) are defined at the micro-scale
3. The equilibrium equation couples both macro and micro levels since, although the stresses are defined macroscopically, the constitutive Eq. (29) depends on the micro-structural topology.

### Algorithmic separability

As pointed-out above, the addressed minimization problem in Eq. (47) entails multi-scale coupling and non-linearities. Due to its non-linear character different solutions, corresponding to different local minima, might be expected. Due to the multi-scale coupling character, the computational robustness of the minimization scheme can be seriously affected.

As a first step, a separation of the minimization problem is introduced here to overcome those difficulties. The original problem in Eq. (47) is slightly rephrased as:

48 $$\begin{aligned} \begin{array}{cc} \underset{\sigma }{\text {minimize}} &{} {\left\{ \begin{array}{ll} \begin{array}{cc} \underset{\chi _{\mu }}{\text {minimize}} &{} \int _{\Omega }\sigma :\mathbb {C}_{h}^{-1}(\chi _{\mu }):\sigma \\ \text {subjected to} &{} \frac{\int _{\Omega _{\mu }}\chi _{\mu }}{V_{\mu }}-1\le 0, \end{array}\end{array}\right. }\\ \text {subjected to} &{} \nabla \cdot \sigma =\rho b,\\ &{} \text {+ Boundary conditions.} \end{array} \end{aligned}$$

This subtle change could be thought as a different notation of the same problem or, even more stimulating, a way of solving the problem. Once in that stage, and inspired by the divide and conquer approach, a tentative second step consists of solving the minimization problem locally, i.e., rewriting Eq. (48) as,

49 $$\begin{aligned} \begin{array}{cc} \underset{\sigma }{\text {minimize}}{\displaystyle \int _{\Omega }} &{} {\left\{ \begin{array}{ll} \begin{array}{cc} \underset{\chi _{\mu }}{\text {minimize}} &{} \sigma :\mathbb {C}_{h}^{-1}(\chi _{\mu }):\sigma \\ \text {subjected to} &{} \frac{\int _{\Omega _{\mu }}\chi _{\mu }}{V_{\mu }}-1\le 0, \end{array}\end{array}\right. }\\ \text {subjected to} &{} \nabla \cdot \sigma =\rho b,\\ &{} {\text {+ Boundary conditions.}} \end{array} \end{aligned}$$

where the main change is the exchange between the minimization and the integral operator. Note that the equilibrium equation plus the boundary conditions are solved as a standard FEM equilibrium problem, that is,

50 $$\begin{aligned} K(\chi _{\mu })u= & {} F. \end{aligned}$$

Due to the fact that all unknowns and constraints of the minimization subproblem are defined locally at Eq. (48) and that the cost function ($\int _{\Omega }\sigma :\mathbb {C}_{h}^{-1}(\chi _{\mu }):\sigma )$ is the sum of local positive-definite cost functions ($\sigma :\mathbb {C}_{h}^{-1}(\chi _{\mu }):\sigma \geqq 0)$, the exchange can be done without altering the global solution. In other words, the micro-scale topologies that provide the minimum global (structural) compliance. are those micro-structure topologies leading to minimal local compliances for every local RVE.

### Algorithmic complexity 

Once problem (49) is stated, its algorithmic complexity (here understood as the number of operations to be performed) is tackled .

Considering a typical FEM discretization involving, say, *n* finite elements at every scale (47)  design variables ( values of the characteristic function, $\chi _{\mu }$, at the RVE times  macroscopic sampling points) are involved. Hence, one optimization problem with a grand total of  design variables has to be solved.

In contrast, problem (49), may be seen as a  *local design-variable optimization problem, solved*  *times* (one for every macroscopic sampling point). This makes an enormous difference in terms of the problem complexity and the corresponding computational cost.

The local optimization problem is then sought, as iteratively solving the global equilibrium equation, starting with a given initial micro-structure topology distribution, and modifying it by the algorithm (40) leading to a new RVE topology (in an uncoupled way from the other RVE’s), until convergence. A similar approach is presented in [14].

In addition, a natural strategy to tackle the problem in (49) consists of solving the local optimization problem at all the  macroscopic sampling points in an way uncoupled from each other, which makes the corresponding algorithm highly parallelizable.

## Vademecum-based computational cost reduction

Despite resorting to parallel computation, problem (49) still exhibits high complexity and it becomes computationally unaffordable for real-life problems. In this scenario, a more efficient approach is proposed here. The main idea consists of optimizing “a priori” a very large discrete-set of micro-structures, in the set of possible macro-stresses acting on the RVE, leading to the so-called “Material Catalogue” or “Computational Vademecum” [17]. Then, when a certain optimal microstructure topology is required in the global (multi-scale) design problem a certain optimal microstructure topology is requested, for a given stress-state at the macro-scale sampling point, the Vademecum is consulted and the closest optimal solution is extracted.

More precisely: given the mechanical properties of the base-material, the expensive computations requested for the Vademecum construction are done once-for-all, in an offline process, and the Vademecum outputs (typically the tangent constitutive operator, $\mathbb {C}_{h}$, solution of Eq. (42) and the compliance are stored in a data-base for, a sufficiently large, discrete set of entries ${ \sigma }.$

The actual multi-scale material design problem is then performed “on-line”, and it only involves a recursive equilibrium analysis at the macro-scale combined with *consultations to the Vademecum*. This translates into an impressive reduction of the computational cost of the on-line material design process. It is highlighted that the Vademecum remains the same for a given base-material, disregard the kind of macro-scale structural problem aimed at being optimized.

### Parametric domain

The success of the proposed Vademecum-based strategy crucially relies on the appropriate determination of the Vademecum entries so that a good balance of the error/computational-cost is achieved. Indeed, since the error is produced by the closest-entry strategy, a higher number of entries lowers the resulting error but increases the computational cost related to the construction of the Vademecum.

The parametric domain defines the range of the space of all possible macroscopic stresses $\sigma$. Inspection of Eq. (49) shows that the modulus of $\sigma$ does not play any role in the determination of the optimal RVE topology. In fact, it can be readily proven that

51 $$\begin{aligned} \chi _{\mu }= & {} \text {arg}\left\{ \begin{array}{cc} \underset{\chi _{\mu }}{\text {minimize}} &{} \sigma :\mathbb {C}_{h}^{-1}(\chi _{\mu }):\sigma \\ \text {s.t.} &{} \int _{\Omega _{\mu }}\chi _{\mu }=V_{\mu } \end{array}\right\} \nonumber \\= & {} \text {arg}\left\{ \begin{array}{cc} \underset{\chi _{\mu }}{\text {minimize}} &{} \frac{\sigma }{||\sigma ||}:\mathbb {C}_{h}^{-1}(\chi _{\mu }):\frac{\sigma }{||\sigma ||}\\ \text {s.t.} &{} \int _{\Omega _{\mu }}\chi _{\mu }=V_{\mu } \end{array}\right\} \end{aligned}$$

Therefore, ${\frac{\sigma }{||\sigma ||}}$ is the actual Vademecum entry. For 2D cases the relevant entry space is then made of unit-modulus stress vectors, which lie in unit-radius sphere and can be parametrized in terms of the two Euler angles, $\phi$ and $\theta$, and as:

52 $$\begin{aligned} \sigma =\left[ \begin{array}{c} \sigma _{x}\\ \sigma _{y}\\ \sigma _{xy} \end{array}\right] =\left[ \begin{array}{c} \text {cos}(\phi )\text {cos}(\theta )\\ \text {sin}(\phi )\text {cos}(\theta )\\ \text {sin}(\theta ) \end{array}\right] \end{aligned}$$

**Fig. 7:**
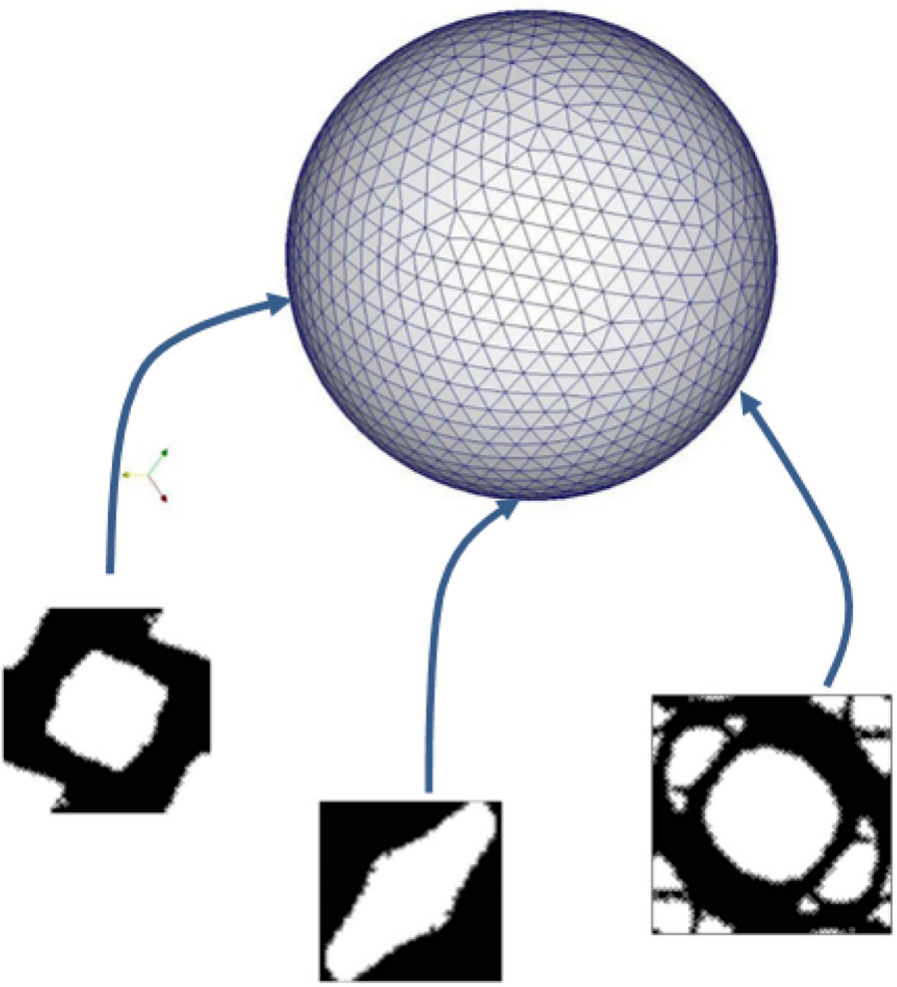
The unit-radius spherical parametric domain (Computational Vademecum)

Hence, the parametric domain is represented by the unit radius sphere. Each point of the sphere can be seen as a micro-structure optimization case, which returns some homogenized elastic properties associated to an optimal topology (see Fig. 7).

For the subsequent examples the sphere has been discretized by means of a structured mesh of 16386 points. However, the bottom half points need not to be computed because they have an homologous point at the top half of the sphere. This is because $\sigma$ or $-\sigma$, plugged in problem (51), result in the same objective function. Besides, only a quarter part of the top half of the sphere must be computed due to symmetries and mirroring. Consequently, an eighth of the whole sphere has been actually considered for the computations, resulting into 2145 points to be actually computed.

As for the involved computational cost: it is remarked that the Vademecum computation is fully parallelizable for the different entries (every case is fully uncoupled), and, therefore, the algorithm is “embarrassingly parallel” in clusters of distributed memory architecture.

In the examples presented in this section, a cluster of 200 workers (Intel Xeon E5) has been used to compute around 2000 entries of the Vademecum, for a given volume fraction. The resulting computational time was around 3h.

**Fig. 8:**
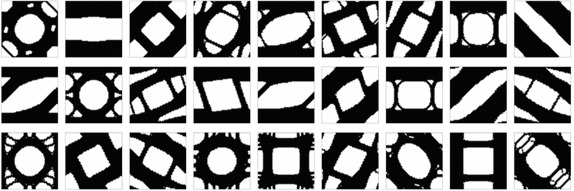
Typical micro-structure topology outputs of the Computational Vademecum

Some examples of this optimal RVE topologies can be seen in Fig. 8. They have been computed for the solid volume fraction at the RVE $V_{\mu }=0.6$. Note that no length scale control scheme is imposed, thus, thin and non manufacturable topologies might appear. Perimeter penalization, see [25], or filtering techniques might be applied as potential remedies.

It is worth mentioning that construction of such Vademecum requires a very robust methodology for the RVE topological design, so that none of the desired entry points fails to be computed. In this sense it has to be remarked that the use of the topological derivative concept and its application in the algorithm (40), as proposed in this work, fulfills this requirement. All cases converged for a constant value of the penalty, with a convergence tolerance ${ \theta <1^{o}}$ and a tolerance on the volume constraint $\text {TOL}<0.001$.

It is also remarkable from Fig. 8, that, for many cases, the obtained optimal topologies are far from being intuitive.

**Fig. 9:**
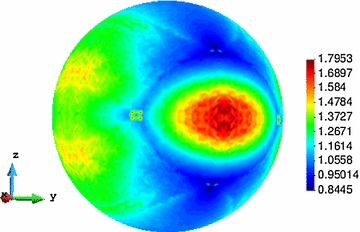
Optimal compliance values over the parametric domain

Associated to the optimal topologies, and based on problem (51), the corresponding RVE compliances for every point of the parametric space are computed, and they are displayed in Fig. 9. It has to be emphasized that, from the theoretical point of view, there is no guarantee that a local minimum has been achieved in all cases, although numerical experiences have evidenced that the obtained compliances are very close to the global minima.

As explained before, the homogenized constitutive operator, $C_{h}$, and the compliance (cost function), constitute the relevant “outputs” of the Vademecum. Indeed, only the compliance is strictly needed in the iterative algorithm. However, the homogenized constitutive operator is also stored in the Vademecun in order to be used for post-processing purposes, when requested, at the end of the analysis.

**Fig. 10:**
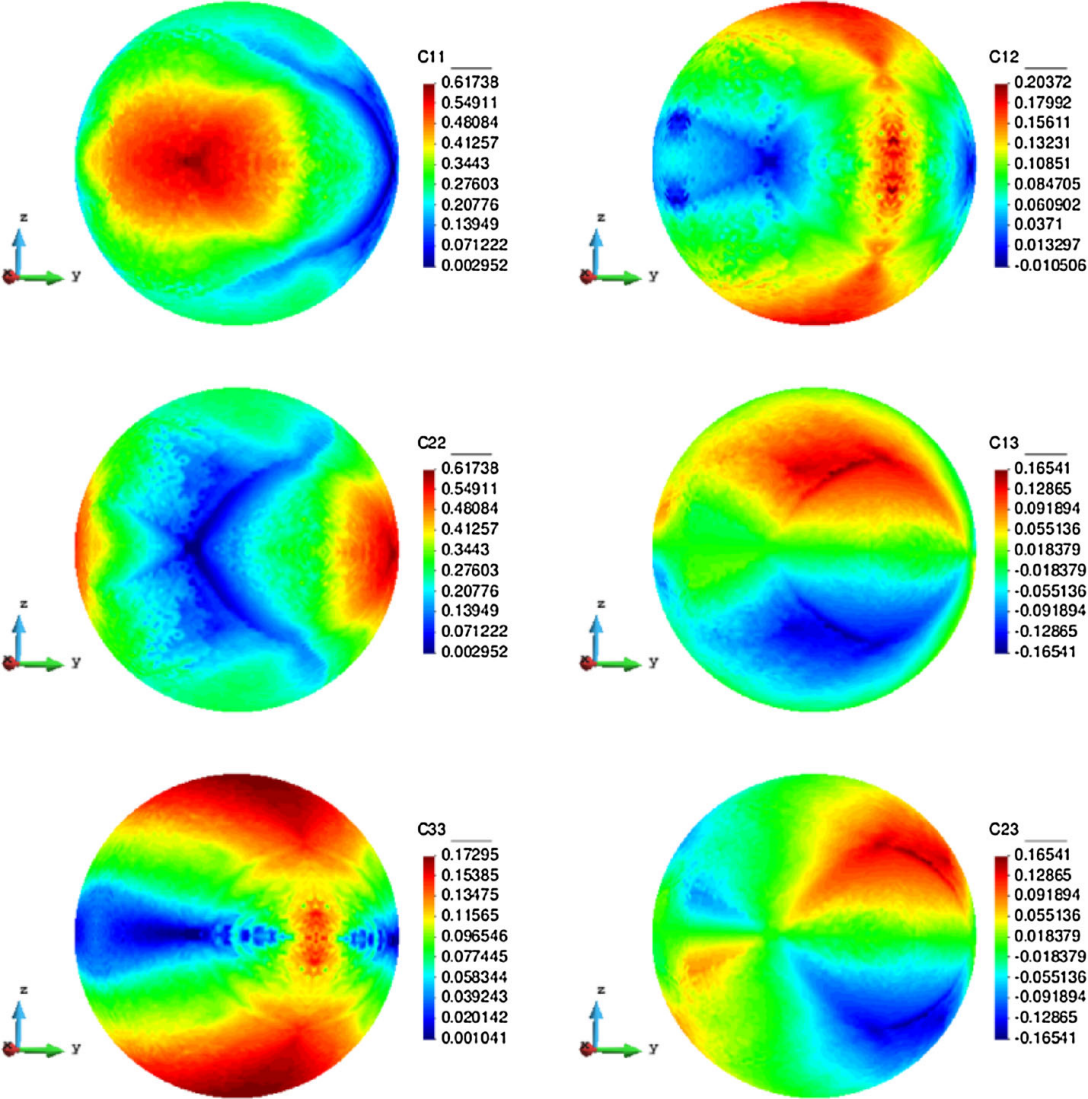
Maps of the constitutive tensor components on the unit-radius spherical parametric domain

Accordingly, in Fig. 10, the Vademecum outputs for the optimal homogenized components of $\mathbb {C}_{h}$ are presented.

There, the major symmetries of $\mathbb {C}^{h}$ (symmetric character of the maps of the symmetric components) as well as the rotated mirroring $\mathbb {C}_{11}$– $\mathbb {C}_{22}$ and $\mathbb {C}_{13}$– $\mathbb {C}_{23}$ can be observed.

## Numerical algorithm

In the following the numerical algorithm, implemented in a Matlab code, for solving the minimization problem (49) is presented.

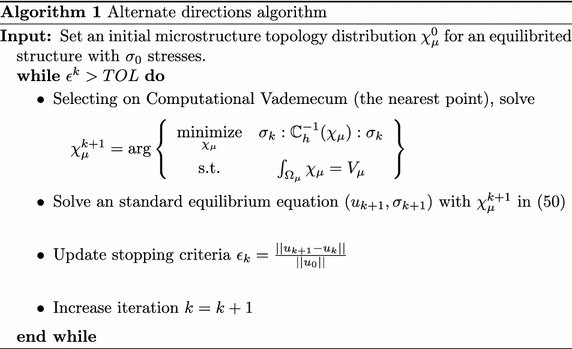



The described algorithm stems naturally from Eq. (49). It is based on the alternate directions scheme, extensively used in the literature (see for example [12]). Since linear elasticity is considered in the mechanical stage, the stresses are obtained by solving a linear problem, using the topology-updated stiffness-matrix. Then, in the subsequent optimization stage, by using the closest entry to the obtained stresses, the optimal mechanical properties, and the corresponding topology, are obtained by consultation of the material catalog (Vademecum). Moreover, the non-linear character of the optimization problem is already accounted for in the Computational Vademecum construction (offline).

As for the global coupled problem, the non-linearity is accounted for by the alternate directions (fixed point) algorithm, and, therefore, only a linear convergence ratio is expected. In spite of this, “Numerical examples” section, it is shown that only few iterations are required for solving this non-linearity.

## Numerical examples

**Fig. 11:**
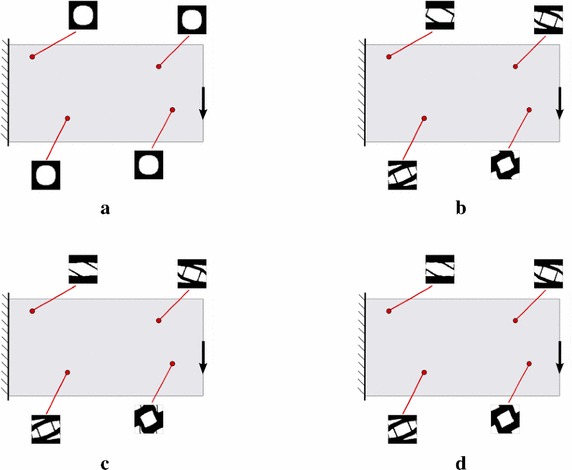
Cantilever beam microstructure design: RVE topology distribution along the iterative design process. **a** Iteration 1, **b** Iteration 2, **c** Iteration 3, **d** Iteration 4

In order to assess the proposed approach some numerical examples are presented next. In all cases the solid volume fraction at the RVE is $V_{\mu }=0.6$, thus satisfying $\int _{\Omega _{\mu }}\chi _{\mu }=V_{\mu }=0.6$. As start point in the iterative procedure, a micro-structure with a centered circular void fulfilling this condition is taken at all RVE’s (see Fig. 11a).

### Cantilever beam

The microstructure topological design of the cantilever beam in Fig. 11 is studied. The dimensions are 2 m length $\times$ 1 m height, and plane stress conditions are assumed. The beam is loaded by a unit vertical point force, at the right end center, and it is clamped at the left end.

This rectangular macroscopic domain, is discretized into 2618 three-noded triangular elements. The elastic properties of the basis material are: Young modulus $E_{\mu }=1$ and Poisson ratio $\nu _{\mu }=0.3$. In Fig. 11 the evolution of the micro-structure topology, along the iterative design process are displayed.

**Fig. 12:**
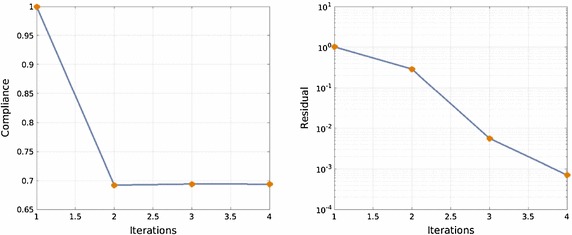
Cantilever beam microstructure design: compliance and residue evolutions for the alternate directions algorithm

**Fig. 13:**
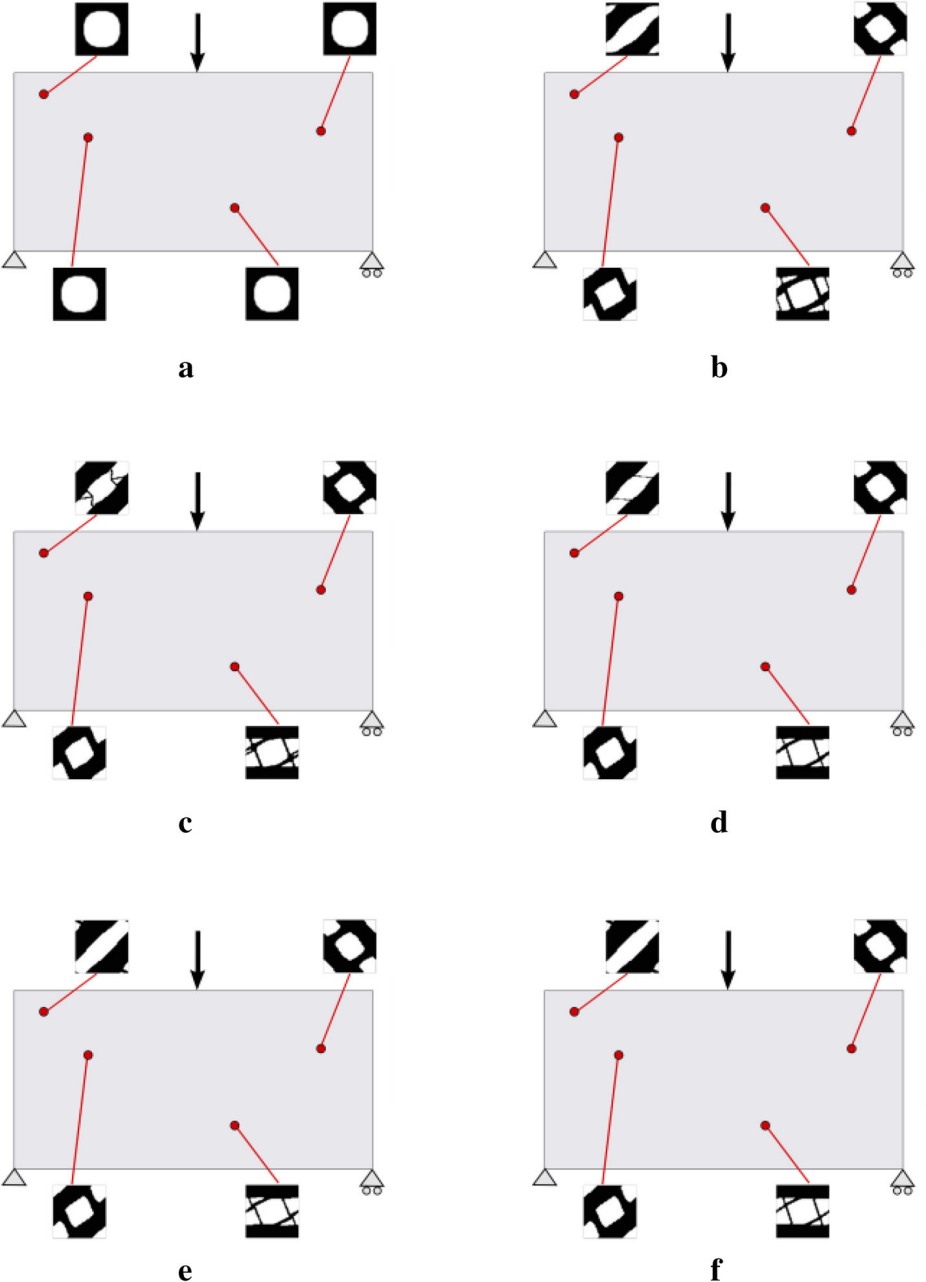
Simply supported bending beam: RVE topology distribution along the iterative process. **a** Iteration 1, **b** Iteration 2, **c** Iteration 3, **d** Iteration 4, **e** Iteration 5, **f** Iteration 6

In Fig. 12, the evolution of the global cost function (structural compliance) and of the residue of the alternate directions algorithm is depicted. As it can be observed in Fig. 12, four iterations suffice to achieve full convergence with a 30 % reduction of the original compliance. The convergence ratio of the iterative process is linear, as expected from the used alternate directions algorithm.

### Bending beam

The microstructure topological design of a simply supported bending beam is studied. The same macroscopic domain as in the previous example, is now discretized in 5056 linear triangular elements. The beam is loaded with a vertical unity force at the mid-span (see Fig. 13).

**Fig. 14:**
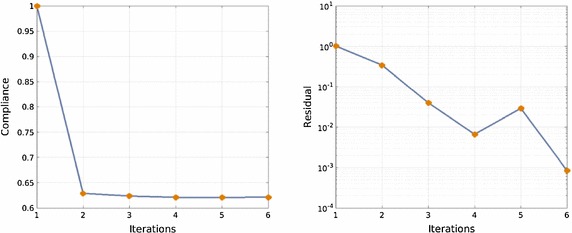
Simply supported bending beam: compliance and residue evolutions for the alternate directions algorithm

The evolving micro-structure distribution during the iterative design process is depicted in Fig. 13. In Fig. 14 it can be observed that a fast convergence is achieved also in this case (six iterations) leading to almost 40 % reduction of the structural compliance.

## Concluding remarks

In this work a new approach to computational material design has been presented and applied to multi-scale topological design of structural materials. A computational homogenization scheme for two scales (FE2) has been considered to account for the impact of the lower scale material distribution on the global structural properties. This is done through the resulting point-wise homogenized elastic properties.

In this context, a through-scale optimization problem has been introduced, where the cost function (compliance) is defined at the structural scale, whereas the design variables (the values of the characteristic function at the RVE) are defined at the micro-scale. This is a challenge which is faced in an innovative way in this work.

To overcome the exorbitant cost typically encountered in brute force FE2 homogenization methods, and taking advantage of the macro/micro separability properties of the stated problem, a Vademecum-based strategy has been devised. It consists of the, off-line, construction of a discrete material catalog, containing an appropriate large set of optimal RVE designs, each one corresponding to a single macroscopic stress state. The topological derivative mathematical tool, jointly with a level set algorithm, based on “slerp” interpolations and a specific line search method, have proved robust enough to provide the optimal RVE designs necessary for the Vademecum construction (more than 2000 for the presented examples). Once this Vademecum is available, it can be repeatedly consulted for the optimal material design of any structure made of the same base material.

For the examples presented in this work, a prescribed volume fraction, has been considered in the construction of the Computational Vademecum. Naturally, the Vademecum computation could also be extended to account for a range of volume fractions, this not implying any conceptual change in the computations, other than the extension of the Vademecum parametric domain with an additional dimension (the volume fraction).

However, the use of this extended Vademecum in a context where the volume fraction may vary from point to point (e.g. when optimizing the weight of the macroscopic structure) is not trivial and it is left for future works.

In this framework a global optimization algorithm, based on consultation of the Vademecum in combination with the equilibrium solution of the macroscopic structural problem, results into a highly non-linear problem that, however, can be solved by means of a fixed point algorithm in remarkably robust fashion. by means of a fixed point (or alternate directions) algorithm.

The resulting strategy becomes both robust and computationally efficient for tackling the microstructure computational design problem in structural materials. Some representative examples illustrate the proposed methodology, and show that large reductions of the structural compliance (up to 40 %) can be achieved by using the proposed “local” topology design strategy, in front of the alternative “homogeneous” material distribution.

It is worth mentioning that, although a smooth transition of material distribution is often seen by the authors, some incompatibilities could appear. Additional constraints must be imposed in order to avoid manufacturability limitations. In our current ongoing work, this issue is addressed and an improved methodology will be proposed.

The issue of the topological continuity/compatibility at micro-scale points has also to be commented. In fact, it has to be remarked that a point-to-point varying topology, as considered here, is an idealized situation, which is not manufacturable in practice and, therefore, it has little industrial interest.

In realistic cases, a piecewise constant topological design, so that the same topology is kept constant in specific macroscopic parts (or components), has much more interest. The extension of the here proposed approach to these cases is going to be presented in a forthcoming work.

Some other important issues remain open in the setting of computational material design. Typically: (1) manufacturability issues, related to piece-wise design of the macrostructure in homogeneous components, and (2) simultaneous topological design, at the macro and micro scales. They are object of ongoing research, and they will be presented by the authors in future works.
